# Anesthetic management of inguinal hernia in an ex-premature infant with subglottic stenosis: a case report

**DOI:** 10.1186/s40981-023-00652-6

**Published:** 2023-09-13

**Authors:** Yumi Doi, Satoshi Ekuni

**Affiliations:** 1 Department of Anesthesia, Takatsuki General Hospital, 1-3-13 Kosobe-Cho, Takatsuki, Osaka 5691192 Japan; 2 Pediatric Perioperative Center, Takatsuki General Hospital, 1-3-13 Kosobe-Cho, Takatsuki, Osaka 5691192 Japan

**Keywords:** Subglottic stenosis, Inguinal hernia, Regional anesthesia, Treatment strategy, Rigid bronchoscopy, Spontaneous respiration

## Abstract

**Background:**

We report the anesthetic management of inguinal hernia repair for an infant with subglottic stenosis. A previously scheduled operation had been cancelled due to unexpected airway trouble during the induction.

**Case presentation:**

A boy was born at 24 weeks of gestation and his trachea was intubated for 45 days. At 16 months old, surgery for inguinal hernia was planned, but cancelled due to unexpected narrow airway, and subglottic stenosis was first suspected. At 17 months old, he was transferred to us for inguinal hernia surgery. After careful discussion between the surgical team and the anesthesiologists, a strategy to manage this patient was developed. He underwent open hernia surgery under spinal anesthesia and diagnostic rigid bronchoscopy under tubeless general anesthesia separately, which revealed low-grade stenosis and some subglottic cysts. The postoperative course was uneventful.

**Conclusion:**

Interdepartmental discussion weighing risks and benefits may deduce the safest and most appropriate anesthesia method.

## Background

Subglottic stenosis (SGS) can develop in patients with a history of intubation, especially in prematurely born infants [1]. A patient with mild SGS may be undiagnosed due to inconspicuous respiratory symptoms. We report the anesthetic management of an infant boy with SGS that was scheduled for right inguinal hernia repair under central neuraxial block and airway evaluation under general anesthesia with spontaneous breathing. A previous operation elsewhere had been cancelled due to unexpected airway trouble during induction of general anesthesia.

## Case presentation

A 17-month-old ex-premature boy with SGS, weighing 6.8 kg, was referred from another hospital for surgical repair of right inguinal hernia due to challenging airway management.

He was born prematurely at 24 weeks and 0 days of gestation, weighing 488 g. His trachea was intubated for 45 days after birth. When his trachea was re-intubated for 2 days for the treatment of retinopathy of prematurity on the 81st day after birth, extubation failed. He was kept intubated for a further 7 days, but SGS was not suspected at that time. After a second attempt of extubation, non-invasive ventilation was performed for 4 weeks before the patient was discharged to home with oxygen therapy of 0.25 L·min^−1^ by nasal cannula.

**Fig. 1:**
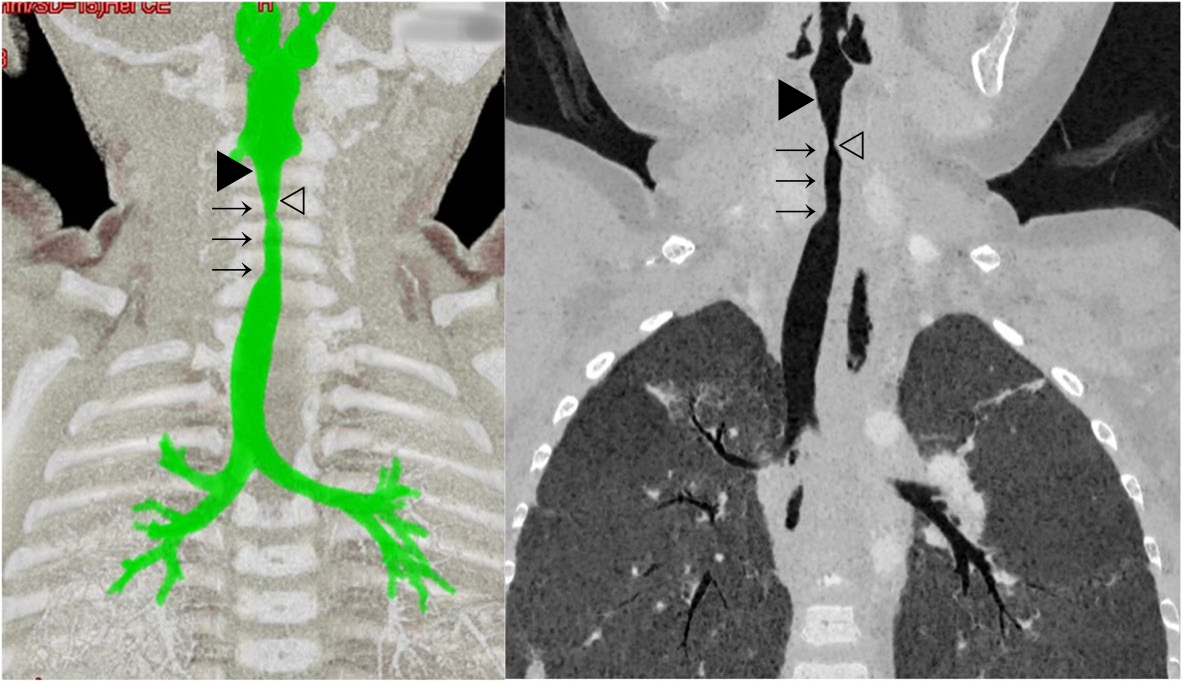
CT images. The length of stenosis (arrow) was 1.2 cm. Stenosis started at approximately 7.5 mm below the vocal cords (black triangle). At the narrowest part (white triangle), the anterior-posterior diameter was approximately 4.0 mm and the transverse diameter was 1.7 mm. Below the stenosis, the diameter of the trachea was 7 mm, which is normal

Surgery was initially scheduled at another hospital at the age of 16 months. During the induction of anesthesia, however, the anesthesiologists unsuccessfully attempted intubation with a 3.5-mm inner diameter uncuffed endotracheal tube (ETT) and a 3.0 mm inner diameter uncuffed ETT several times. A 2.5-mm ETT was successfully intubated, but the surgery was cancelled due to the unexpectedly narrow airway, which was a concern of SGS. The patient was admitted to the intensive care unit, and the ETT was removed the next day. Severe stridor and retraction were noted, so SGS was suspected for the first time. CT scan one week after extubation revealed 1.2-cm-long SGS and the narrowest diameter of 1.7 mm in the subglottic area (Fig. 1). The patient was referred to our hospital for further management.

On admission, his percutaneous oxygen saturation was 98–100% on nasal cannula of 0.25 L·min^−1^ oxygen. He showed slight retraction, but there was no stridor on rest. When he cried, subcostal retraction worsened and slight stridor was auscultated around the neck. His respiratory status was much better than we had heard from the previous doctors. We presume that the previous hospital took the CT scan when there were remaining effects of tracheal intubation in the subglottic area. After transfer, we started inhaled steroids and there was gradual improvement of his condition.

Primary concerns regarding the patient at that time were repeated incarceration of inguinal hernia and respiratory distress. After discussion between the anesthesiologists and the surgical team, we concluded that rigid bronchoscopy (RBS) could wait and we developed three potential strategies to manage the patient (Table 1). In Discussed Option #3 (Table 1), the surgery could be performed under spinal anesthesia and sedation [2, 3], and then RBS maintaining spontaneous respiration could be performed on another day. This would mean intubation would be unnecessary, and there would be no invasion to the subglottic area at all. Due to the complexity of the patient’s medical history (ex-premature and repeated incarceration of inguinal hernia), the procedure could take longer, so epidural anesthesia was planned to be combined with spinal anesthesia [4].

**Table 1:** Advantages and disadvantages of the treatment strategy and anesthesia methods

	Surgical plan/anesthesia methods (airway management)	Pros	Cons
Discussed Option #1	Laparoscopic inguinal hernia repair and RBS	- All performed at once - Visualization of ipsilateral hernia	- Intubation necessary for laparoscopic surgery - Difficulty of extubation, or tracheostomy possibility - CO_2_ insufflation for ex-prematurity with CLD
	Intubation after RBS + PNB		
Discussed Option #2	Open hernia repair and RBS	- All performed at once - Intubation unnecessary	- Intubation possible, depending on the degree of subglottic stenosis - No necessity of GA for open surgery
	SGD or Intubation after RBS + PNB		
Discussed Option #3	Open hernia repair first, and RBS on another day	No invasion of subglottic area	- Two-time anesthesia - Longer hospital stay
	Spinal anesthesia under sedation at first time and spontaneous respiration at second time		

*RBS* Rigid bronchoscopy, *PNB* Peripheral nerve block, *CLD* Chronic lung disease, *SGD* Supraglottic airway device, *GA* General anesthesia

**Fig. 2:**
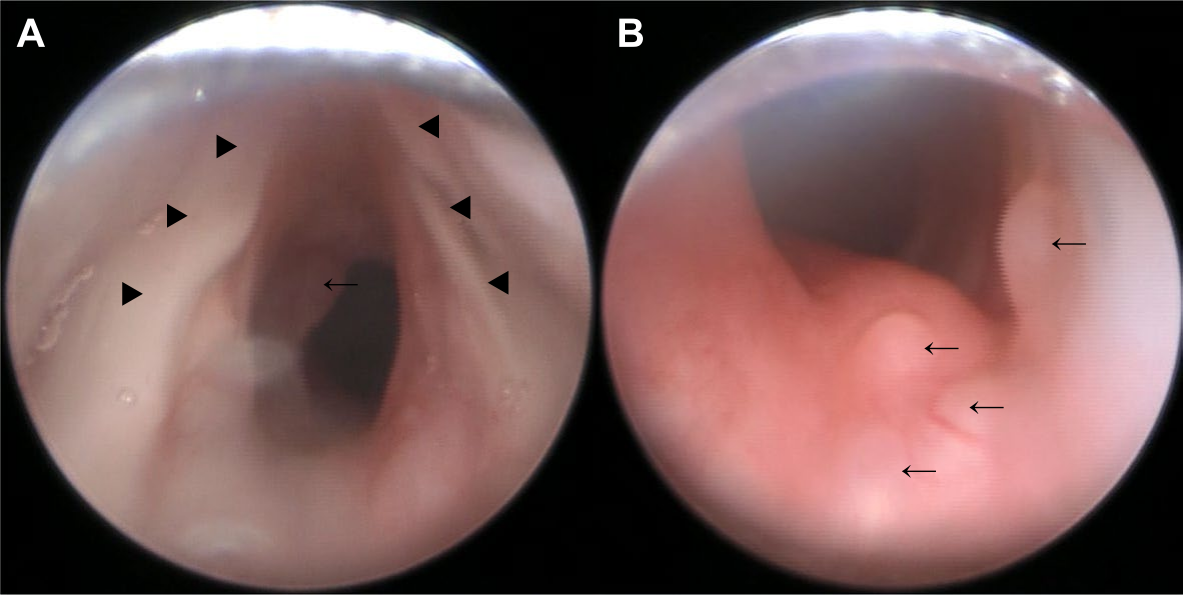
Rigid bronchoscopy images. Spontaneous respiration was maintained during rigid bronchoscopy. **A** The degree of subglottic stenosis was Myer-Cotton classification grade I. **B** Some subglottic cysts (arrow) were observed in subglottic area. Black triangle: vocal cord

After standard monitors were applied and a peripheral intravenous line was established, 0.01 mg·kg^−1^ of atropine was given and then dexmedetomidine was administrated at the rate of 0.8 mcg·kg^−1^·h^−1^ for 5 min and up to 1.2 mcg·kg^−1^·h^−1^ for 14 min before spinal tap. Spinal anesthesia was performed with a 25-gauge needle at L4/5 (0.15 ml·kg^−1^ of 0.5% hyperbaric bupivacaine) in right lateral position and a caudal epidural catheter was inserted via the sacral hiatus without any difficulty under light sedation. Dexmedetomidine was continued between 0.4 and 1.2 mcg·kg^−1^·h^−1^, depending on the patient’s respiratory status and the depth of sedation until the end of surgery. Hemodynamic and respiratory statuses were stable, and the patient was appropriately sedated. Operators were satisfied with sufficient infant immobility to allow satisfactory completion of the operation. The operation time was 61 min, and the anesthesia time was 105 min. The postoperative course was uneventful. Six days after the hernia repair, RBS was performed under general anesthesia maintaining spontaneous respiration, using propofol, ketamine, and topical anesthesia to the larynx. Low-grade stenosis and some cysts were revealed in the subglottic area (Fig. 2). Those lesions were considered to not require immediate further intervention except continued inhaled steroid treatment and long-term observation.

## Discussion

The key to successful management in the present case was the multidisciplinary collaboration between the anesthesia and surgical teams to develop the treatment strategy.

The anesthesia method for inguinal hernia repair differs depending on the surgical procedure [2, 5]. There are two options, laparoscopic surgery or open surgery, each with their own advantages and disadvantages, including modes of anesthesia [6, 7]. The treatment strategy is often decided before appropriate discussion with the anesthesiologist to elucidate their perspective, even in cases in which there seem to be anesthetic problems. A tailored approach is recommended, taking into account the expertise of both the anesthesia and surgical team members [8].

In our hospital, infant cases of inguinal hernia are usually treated by laparoscopic surgery under general anesthesia with tracheal intubation and peripheral nerve block (Discussed Option #1, Table 1). As part of the discussion regarding this patient, disadvantages of laparoscopic surgery were noted, including consideration of the necessity of intubation and CO_2_ insufflation because he had suspected SGS and chronic lung disease (CLD). Our surgeons preferred laparoscopic surgery under general anesthesia with tracheal intubation because surgery for this patient would be time consuming and difficult because he was born premature and had a history of repeated incarceration. Also, there are advantages of laparoscopic surgery, which can identify and treat a contralateral inguinal hernia occurring in ex-premature infants. If Discussed Option #1 (Table 1) was performed according to the surgeons’ initial preference, there were risks of difficult ETT removal, and concerns regarding respiratory management under intubation postoperatively and PICU admission. In the worst case scenario, this could result in the need for tracheostomy. As a result of discussion, it was decided that avoiding tracheal intubation should be prioritized.

If surgeons elect for open surgery as a procedure, spinal anesthesia is thought to be a good option because tracheal intubation can be avoided. If airway evaluation and open surgery are planned to be performed at the same time (Discussed Option #2, Table 1), the RBS should be performed under general anesthesia first, and the airway may be secured with a supraglottic device, or ETT, depending on the findings of subglottic lesions. Performing the surgery and airway evaluation simultaneously nullifies the advantage of choosing open surgery, because the open surgery does not require general anesthesia in the first place.

In Discussed Option #3 (Table 1), the patient would require anesthesia twice and a longer hospital stay. However, spontaneous breathing can be preserved in open hernia surgery under spinal anesthesia and RBS, so it is highly likely that tracheal intubation can be avoided. Considering the history of intubation in the early stages of our patient’s life and difficult insertion of age-appropriate size of ETT at the induction of previous anesthesia, and with due consideration of CT findings and the patient’s physical symptoms, it was decided to be important to avoid tracheal intubation, despite the degree of SGS being mild [9]. We concurred that Discussed Option #3 was the best strategy.

The true incidence of acquired SGS and cysts and intubation-related laryngeal injury is difficult to assess. The duration of intubation is a significant risk factor [10]. Unless there is a strong suspicion of SGS, the evaluation is not always performed, even when the patient has a history of intubation or premature birth. Outpatient follow-up is usually performed by neonatologists or pediatricians, but their low awareness of SGS because of low prevalence may also contribute to the low awareness of anesthesia-related risks [11]. Even with persistent respiratory symptoms and a history of long-term intubation, SGS may be misdiagnosed as CLD because symptoms of SGS may mimic the characteristic features of CLD [12] and because of the history of premature birth often accompanies CLD. Anesthesiologists who care for children with such medical histories and respiratory symptoms must therefore be cautious in their preoperative physical evaluation.

We provided a secure perioperative strategy to perform inguinal hernia surgery and airway assessment in an infant whose surgery had been cancelled at another hospital due to SGS. The patient underwent open hernia surgery under spinal anesthesia and diagnostic RBS under tubeless general anesthesia separately. No tracheal intubation was performed in either operation. Discussion weighing risks and benefits may deduce the safest and most appropriate anesthesia method.

## Data Availability

Not applicable.

## Abbreviations

SGS: Subglottic stenosis
ETT: Endotracheal tube
RBS: Rigid bronchoscopy
CLD: Chronic lung disease
